# The aleurone layer of cereal grains: Development, genetic regulation, and breeding applications

**DOI:** 10.1016/j.xplc.2025.101283

**Published:** 2025-02-12

**Authors:** Huawei Liang, Jian Zhou, Chen Chen

**Affiliations:** 1 Jiangsu Key Laboratory of Crop Genomics and Molecular Breeding/Zhongshan Biological Breeding Laboratory/Key Laboratory of Plant Functional Genomics of the Ministry of Education, Agricultural College of Yangzhou University, Yangzhou 225009, China; 2 Jiangsu Co-Innovation Center for Modern Production Technology of Grain Crops, Agricultural College of Yangzhou University, Yangzhou 225009, China; 3 Yangzhou Modern Seed Innovation Institute, Gaoyou 225600, China

**Keywords:** cereal, aleurone layer, cell differentiation, nutritional quality, molecular regulation, breeding application

## Abstract

Cereal aleurone cells are differentiated from triploid endosperm cells and exhibit distinct cytological, physiological, and biochemical characteristics that distinguish them from the starchy endosperm cells of cereals. Aleurone cells maintain viability throughout seed development, whereas starchy endosperm cells undergo programmed cell death during maturation. Despite variations in aleurone-related traits among cereal species, the aleurone layer plays a crucial role in regulating many aspects of seed development, including the accumulation of storage reserves, the acquisition of dormancy, and germination. Given that many nutrients—such as lipids, dietary fibers, vitamins, and minerals like iron and zinc—are predominantly accumulated in the aleurone cells of cereal grains, this layer has attracted considerable attention aimed at improving the nutritional value of cereals. This review provides a comprehensive overview of the developmental, genetic, and molecular basis of aleurone cell differentiation and proliferation. It focuses on the improvement of aleurone-related traits informed by knowledge of the molecular networks governing aleurone development and presents a detailed discussion on the challenges and potential solutions associated with cereal improvement through the manipulation of aleurone-related traits.

## Teaser

Aleurone cells contain substantial amounts of nutrients, including lipids, proteins, dietary fibers, vitamins, and minerals. This review provides a comprehensive overview of the development, genetic regulation, and breeding applications of the aleurone layer. Furthermore, it explores the challenges associated with leveraging aleurone-related traits and suggests potential solutions to address these challenges.

## Introduction

Cereals are the foundation of a healthy diet, providing an important source of calories and essential nutrients for human consumption. A growing body of epidemiologic evidence suggests that whole grain consumption may reduce the risk of cardiovascular diseases, gastrointestinal cancers, and type 2 diabetes, in addition to facilitating body weight regulation (Rosa-Sibakov et al., 2015). The increase in global consumption of refined cereal products could lead to a number of health issues, as whole grains are richer in nutrients than their refined counterparts (Guo et al., 2022). Consequently, many countries are promoting whole grain consumption for its health benefits and potential to reduce the burden on health care systems (Miller, 2020).

A cereal grain is composed of three distinct parts: the endosperm, the embryo, and the surrounding maternal tissues, including the pericarp, the seed coat (testa), and the nucellar epidermis (hyaline layer) (Zhang et al., 2021). Milling and polishing grains result in the removal of the embryo and maternal tissues, along with the outermost portion of the endosperm, known as the aleurone layer. Since nutrients are unevenly distributed within the cereal grain, the resulting bran contains a greater amount of proteins, lipids, minerals, and vitamins than the refined flour, which is characterized by a high starch content (Luh et al., 1991). Notably, a significant portion of the nutrients present in bran are derived from the aleurone. For example, the aleurone layer of wheat (*Triticum aestivum*) is a concentrated source of essential minerals and vitamins, containing approximately 50% of the total dietary fiber and the majority of antioxidants accumulated in the seed (Chen et al., 2024). Therefore, the aleurone is a fundamental basis for the health benefits derived from whole grain products.

Enhancing the nutritional density of cereals through the manipulation of aleurone-related traits has been proposed as a potential breeding strategy (Meziani et al., 2021; Wu et al., 2022). However, while much of the current literature concentrates on the nutritional value of the aleurone, there lacks a comprehensive examination of the practicality of this concept (Atwell et al., 2007; Brouns et al., 2012; Rosa-Sibakov et al., 2015; Aslam et al., 2018; Lebert et al., 2022; Chen et al., 2024). This review summarizes the current progress in the field, with a particular focus on practical strategies for improving aleurone-related traits. It also discusses the challenges associated with these strategies and proposes potential solutions.

## The aleurone layer and its biological significance

**Figure 1:**
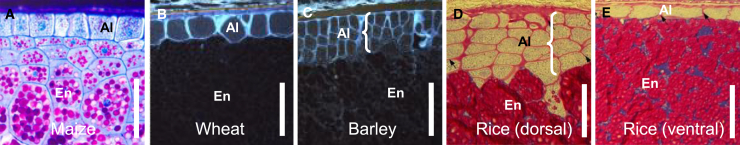
Aleurone layer in different cereals. **(A)** A microscopic section of maize endosperm. Starchy endosperm cells are filled with starch granules stained pink with periodic acid-Schiff (PAS) reagent. Al, aleurone layer; En, starchy endosperm. Scale bar corresponds to 50 μm. Image reproduced and modified from Becraft and Yi (2011). **(B and C)** Autofluorescence images showing the aleurone layer in wheat **(B)** and barley **(C)**. Images were collected using UV excitation (330–380 nm) and emission > 420 nm. Scale bars correspond to 100 μm. Images were duplicated and modified from Jääskeläinen et al. (2013). **(D and E)** Overview of rice aleurone layers on the ventra**l (D)** and dorsal **(E)** side of rice caryopsis. Carbohydrates are magenta, stained by PAS. Proteins are blue, stained by Coomassie brilliant blue (CBB). Scale bars correspond to 50 μm. Images were duplicated and modified from Yu et al. (2021).

The aleurone is a distinctive tissue found in the majority of angiosperm seeds. It is composed of one or more outer layers of endosperm cells situated beneath the seed coat. Aleurone cells remain intact throughout the process of seed development and are consistently present in mature seeds (Gontarek and Becraft, 2017). Moreover, the aleurone is the only endosperm cell type that remains viable in fully mature, desiccated seeds. In cereals, aleurone cells are considered triploid endosperm cells; however, they are cytologically distinct from other endosperm cell types (Olsen, 2001; Wu et al., 2016a). In cross-section, the aleurone cells are well-organized, densely populated, and cuboidal in shape (Figure 1). Their thick auto-fluorescent cell walls also feature various inclusions like protein-carbohydrate bodies, aleurone grains, and lipid droplets. Unlike inner endosperm cells, the aleurone does not typically contain starch granules in the mature seed. Consequently, the aleurone and starchy endosperm have distinct biochemical properties. The aleurone is rich in proteins, lipids, vitamins, minerals, and dietary fiber, whereas the endosperm is predominantly composed of carbohydrates. Many cereals also contain a subaleurone layer, which is cytologically and biochemically distinct from the starchy endosperm. This layer consists of a few cell layers and serves as an intermediary between the nutrient-dense aleurone and the carbohydrate-rich starchy endosperm. The subaleurone tissue is much lower in starch content and accumulates a high concentration of storage proteins. For example, the subaleurone layer in rice (*Oryza sativa*) typically consists of four to six layers of cells with smaller starch granules and is notable for its abundance of protein bodies and lipid bodies (Wu et al., 2016b).

**Figure 2:**
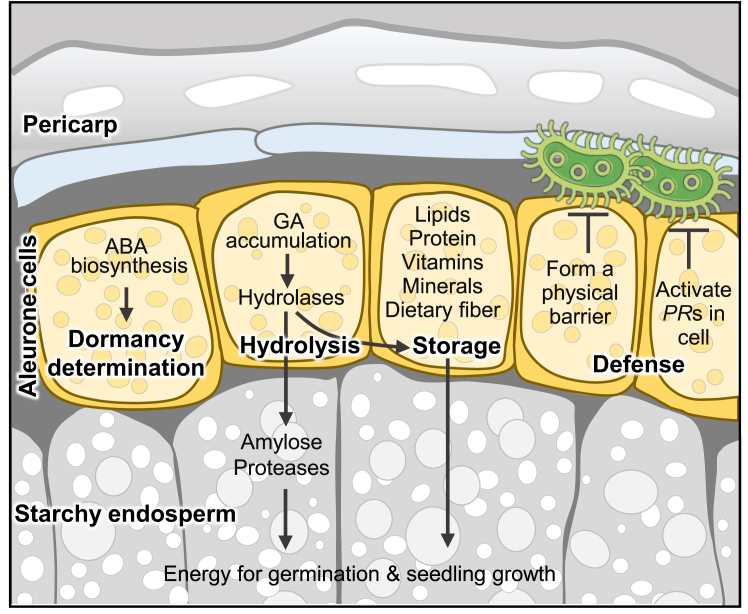
Biological significance of the aleurone layer. This diagram illustrates the roles of aleurone cells during development to ensure seed maturation and germination. During the filling stage, the aleurone cells store many nutrients, including lipids, proteins, minerals, and vitamins. During germination, the aleurone layer acts as a primary determinant of seed dormancy due to the accumulation of the phytohormone abscisic acid (ABA) in the cells. The aleurone cells form a defensive barrier against pathogens throughout seed development. During germination, aleurone cells secrete hydrolases (such as amylase and lipase) induced by gibberellin (GA) to digest the reserves in aleurone and endosperm cells to promote seed germination and seedling growth.

In response to imbibition, the embryo releases the phytohormone gibberellin acid (GA), prompting the aleurone to secrete hydrolases (Bethke et al., 1997), which in turn facilitates the degradation of reserves that have accumulated in the endosperm and aleurone. This induced hydrolysis and mobilization of reserves promote seed germination and facilitate seedling growth (Penfield et al., 2004; Hong et al., 2012). Additionally, the aleurone layer has been identified as the primary determinant of seed dormancy in Arabidopsis (*Arabidopsis thaliana*) (Bethke et al., 2007). The phytohormone abscisic acid (ABA), which is biosynthesized in the aleurone layer, is proposed as responsible for the induction of dormancy (Lefebvre et al., 2006; Lee et al., 2010). The aleurone layer also functions as a peripheral barrier, safeguarding the starchy endosperm cells against pathogen invasion. Previous studies have shown that several defense genes are highly activated in the aleurone cells of rice (Casacuberta et al., 1991; Takafuji et al., 2021). Furthermore, the transcription factor (TF) NAKED ENDOSPERM (NKD) is involved in the differentiation of aleurone cells and regulates the expression of defense-related genes in the aleurone layer of maize (*Zea mays*) (Gontarek et al., 2016). Therefore, the aleurone can be considered a multifunctional structure, playing a role in storage, hydrolysis, defense, and dormancy acquisition to ensure seed development (Figure 2).

## Development of aleurone in cereal plants

Coenocytic endosperms, which result from double fertilization in the majority of angiosperms (Olsen, 2001), arise through nuclear division uncoupled from cytokinesis. Subsequent cellularization and mitotic divisions create an endosperm with multiple cell layers (Xu and Zhang, 2023). The cells in the outer layer exhibit a distinctive division pattern compared to those in the inner layers. Specifically, the outer cells divide primarily in anticlinal and periclinal orientations, whereas the internal cells divide in random planes (Gontarek and Becraft, 2017). The outermost layers of endosperm cells serve as the founder cell population, which subsequently differentiates into aleurone cells soon after the completion of cellularization in various cereal species (Cho, 1956; Opanowicz et al., 2011; Xiong et al., 2013; Wu et al., 2016b).

Although the development of cereal grains is broadly similar across species, there are some notable morphological differences in the aleurone layer. Maize and wheat typically exhibit a single layer of aleurone cells in mature grains, whereas barley (*Hordeum vulgare*) has three layers (Figure 1A–1C) (Olsen, 2004; Jääskeläinen et al., 2013). In rice, the number of aleurone cell layers is site-specific (Figure 1D and 1E), with a markedly thicker aleurone layer on the dorsal side of the seed than on the ventral side (Hoshikawa, 1967; Wu et al., 2016b). Notably, wheat and barley also possess modified aleurone cells, which exhibit cytological features and expression profiles distinct from typical aleurone cells (Drea et al., 2005). This modified aleurone layer, located near the crease region of the grain, serves as a principal transfer tissue in wheat and barley. Despite its close phylogenetic relationship with the Triticeae subfamily, *Brachypodium distachyon* lacks these modified aleurone cells. Moreover, its aleurone layer is unevenly distributed, similar to rice, with a thicker aleurone layer adjacent to the nucellar projection (Opanowicz et al., 2011). Maize features an array of transfer cells at the junction between the funiculus and the ovule (Charlton et al., 1995). These maize transfer cells appear to be functional homologs of the wheat-modified aleurone cells, with the responsibility of transporting maternal nutritional supplies to the developing seed (Hands and Drea, 2012). Notably, *in-vitro*-cultured young maize endosperms are capable of differentiating aleurone cells in the peripheral region but are unable to develop transfer cells (Gruis et al., 2006), suggesting that modified and normal aleurone cells have distinct identities. This is supported by evidence of divergent gene expression processes between modified aleurone/transfer cells and aleurone cells in wheat and maize (Drea et al., 2005; Pfeifer et al., 2014; Zhan et al., 2015).

## Plasticity of aleurone development

**Figure 3:**
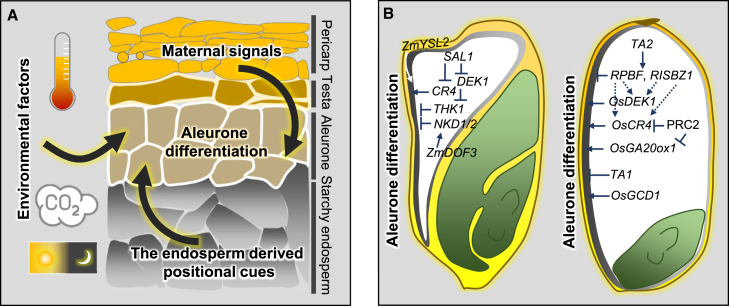
Environmental and genetic regulation of aleurone cell development in cereals. **(A)** Differentiation of aleurone cells is coordinately determined by endosperm-derived positional cues, maternal signals, and environmental factors such as temperature, photoperiod, and atmospheric CO_2_ concentration. **(B)** Genetic regulation of aleurone differentiation in maize (left) and rice (right). In maize, *DEK1*, *CR4*, and *ZmYSL2* positively regulate aleurone differentiation, whereas *THK1*, *SAL1*, and *ZmDOF3* negatively regulate it. *THK1* is downstream of *DEK1* and *NKD1/2* is downstream of *ZmDOF3*. In rice, *OsCR4* and *OsDEK1* promote aleurone differentiation, whereas *RPBF*, *RISBZ1*, *TA1*, and *TA2* inhibit it. *OsCR4* and *OsGA20ox1* are downstream targets of the rice PRC2 complex in the aleurone. Arrows and T-bars indicate positive and negative regulation of aleurone differentiation, respectively.

Aleurone development in cereals demonstrates considerable plasticity in response to fluctuations in the external environment (Figure 3A). For example, Xu et al. (2023a) analyzed the distribution of dorsal aleurone thickness (DAT) in a population of chromosome segmental substitution lines and identified a significant negative correlation between rice DAT and days to heading (Xu et al., 2023b), suggesting that the photoperiod may influence aleurone development. The authors proposed that photoperiod-associated DAT may be due to an indirect thermal effect, as rice lines that head earlier generally coincide with higher temperatures during seed filling (Xu et al., 2023b). This hypothesis is supported by observations that rice varieties cultivated under elevated temperatures exhibited a notable increase in DAT (Nagato and Ebata, 1960, 1965; Nagato et al., 1966; Hoshikawa, 1967). Similarly, the planting season significantly influences rice aleurone thickness. In Japan, early-season rice planting, typically associated with higher temperatures during seed filling, results in increased aleurone thickness compared with seeds from late-season planting (Ahibuya, 1929; Minase et al., 1962; Hoshikawa, 1967).

Research in rice seeds has shown that early-season cropping or high temperatures during seed development increase aleurone-enriched nutrients, such as proteins, lipids, and minerals (Taira et al., 1979; Seo and Chamura, 1980). Conversely, in wheat, exposure to cold stress results in an increase in the protein content of the aleurone layer (Shi et al., 2022). Additionally, nitrogen fertilization levels have been shown to influence the proteins accumulated in the aleurone layer (Minase et al., 1962; Hermans et al., 2021). Elevated atmospheric CO₂ has also been observed to impact aleurone development in the wild rice species *O. meridionalis* (Rahman et al., 2022). It is projected that rising atmospheric CO₂ concentrations could lead to an increase in potential health risks in rice-dependent countries due to a decline in aleurone-enriched nutrients like protein, iron, zinc, and vitamins B1, B2, B5, and B9, in rice seeds (Zhu et al., 2018).

## Position is the key: Determination of aleurone cell fate

Only the outermost peripheral layer of endosperm cells differentiates into aleurone cells (Gontarek and Becraft, 2017). In maize, the inner mitotic daughter cell of an aleurone founder cell differentiates into a starchy endosperm cell (Morrison et al., 1975; Becraft and Asuncion-Crabb, 2000), suggesting that aleurone and starchy endosperm cells originate from the same cell lineages. *In vitro* cultures of maize endosperm cells have demonstrated that the fates of starchy endosperm and aleurone cells are interchangeable, with potential conversion depending on the position of the cells. Specifically, internalized aleurone cells convert to starchy endosperm cells, while starchy endosperm cells at the surface convert to aleurone cells (Gruis et al., 2006). These findings indicate that cell position is the primary determinant of aleurone differentiation. This hypothesis is further supported by the analysis of maize connated kernels, showing that aleurone cells are only present within a limited distance along the fusion plane, whereas starchy endosperm cells are present along the majority of the remaining fusion plane of the twin endosperms (Geisler-Lee and Gallie, 2005). It is therefore proposed that a positional cue induces aleurone differentiation, given that aleurone cells only develop on endosperm surfaces. To date, the precise nature of this positional cue remains unclear (Olsen, 2020). It seems implausible that the cue originates from the maternal tissues, given that cells at the surface can assume aleurone identity following *in vitro* culturing of the starchy endosperm cells (Gruis et al., 2006). There is a general consensus that the positional cue likely originates from within the endosperm. However, in the referenced *in vitro* experiment, the endosperm was excised from developing kernels at 6 days after fertilization (DAF) (Gruis et al., 2006). The aleurone marker *BETL9-like* is expressed as early as 5 DAF, indicating that the aleurone fate is determined by maize at this stage (Royo et al., 2014). It is possible that the surface cells are already marked by the positional cue at this particular stage of development, regardless of whether the cue originated from the maternal cells or the internal cells of the endosperm. Further support for this hypothesis comes from the maize *globby1* (*glo1*) mutant, which develops aleurone-like cells embedded in the inner starchy endosperm cells sporadically (Costa et al., 2003), reinforcing the idea that the signal determining aleurone differentiation in the peripheral endosperm does not originate from the surrounding maternal tissues.

Wang et al. (2004) proposed that minerals accumulated in the cells, which the authors termed "filling wastes," influence the fate of aleurone cell development. Although this hypothesis cannot fully explain results of the *in vitro* endosperm culturing experiment (Gruis et al., 2006), recent research on the maize *shrunken4 (sh4) mutant provides new insights. This study* showed that *sh4* aleurone cells acquired starchy endosperm identities due to a defective YELLOW STRIPE-LIKE oligopeptide metal transporter ZmYSL2 (He et al., 2021). This disruption in cell fate determination in *sh4* was associated with an iron deficiency in the outermost layer of endosperm cells. Considering that minerals accumulated in the seed are transported from the maternal plant, this finding compels us to reconsider the possibility that the positional cues influencing aleurone differentiation may also originate from maternal tissues.

## Genetic regulation of aleurone cell differentiation and division

In addition to environmental factors such as temperature, atmospheric CO_2_ concentration, and photoperiod (Figure 2A), genetic regulation significantly influences aleurone development. Variability in the number of aleurone layers is observed even within a single species. For instance, the DAT of the rice subspecies *Geng* is typically greater than that of *Xian* (Hoshikawa, 1967; Khin et al., 2013). However, the genetic basis for this discrepancy remains to be elucidated. A linkage-based analysis of DAT variation in rice indicates that at least three stable quantitative trait loci (QTLs) are involved. One of these QTLs colocalizes with rice flowering regulators *Heading date 6* (*Hd6*) and *Hd16*, which may account for the observed negative correlation between DAT and days to heading (Xu et al., 2023b). The variability in aleurone thickening in response to elevated temperatures across different varieties (Nagato et al., 1966) suggests that genetic-environmental interactions play a role in the developmental plasticity of rice aleurone. In maize, aleurone typically forms a single layer of cells, whereas some tropical corn landraces exhibit multiple aleurone layers (MALs), ranging from two to nine cellular layers (Wolf et al., 1972; Paulsmeyer and Juvik, 2023; Hong et al., 2024). Previous research has indicated that the MAL trait is controlled by one or two dominant or partially dominant genes (Wolf et al., 1972; Duangploy et al., 1976). A recent study demonstrated that the MAL trait is predominantly determined by a locus on chromosome 8, with several minor loci also contributing in a more additive than dominant manner (Paulsmeyer and Juvik, 2023). In barley, three QTLs governing the number and thickness of aleurone layers were identified using F_2_-F_3_ progeny derived from two varieties with differing aleurone layer numbers (Jestin et al., 2008). These findings highlight the complex genetic regulation of aleurone development in cereals.

Maize kernels can accumulate anthocyanin in the aleurone layer, allowing for the aleurone color to serve as a morphological marker for identifying mutants with aleurone defects. Consequently, most aleurone-related mutants isolated to date are from maize (Table 1). For example, the *defective kernel1* (*dek1*) mutant lacks aleurone, with starchy endosperm cells filling the outer layer (Becraft and Asuncion-Crabb, 2000; Becraft et al., 2002; Lid et al., 2002). Similarly, the *crinkly4* (*cr4*) mutant displays a mosaic aleurone phenotype, with sporadic areas of starchy endosperm cell identity in the periphery (Becraft et al., 1996; Becraft and Asuncion-Crabb, 2000). The *naked endosperm* (*nkd*) mutant exhibits aleurone-less or mosaic aleurone phenotypes, characterized by the presence of multiple layers of peripheral cells that exhibit neither aleurone nor starchy endosperm features (Yi et al., 2015). The *nkd* mutant shows a 15:1 segregation ratio in the F_2_ generation, suggesting that two unlinked recessive genes, *nkd1* and *nkd2*, cause this phenotype (Yi et al., 2015). In contrast, the endosperm of the barley *defective seed5* (*des5*) mutant consists of a monolayer of broader but less dense aleurone-like cells on the periphery, compared to the typical three layers observed in the wild-type (WT) (Olsen et al., 2008). These mutants exhibit a deficiency or absence of aleurone layers, suggesting a positive regulatory role for the underlying genes in aleurone differentiation.

**Table 1:** Genes that regulate aleurone development and differentiation.

Species	Gene name	Locus ID	Functional molecule	Aleurone phenotype of mutant	References
**Maize**	*Defective Kernel1* (*DEK1*)	Zm00001d028818	calpain-type cysteine protease	aleurone-less	Becraft and Asuncion-Crabb (2000); Lid et al. (2002); Becraft et al. (2002)
	*Crinkly4* (*CR4*)	Zm00001d023425	plasma membrane-localized receptor-like kinase	aleurone-less	Becraft et al. (1996); Becraft and Asuncion-Crabb (2000)
	*Naked Endosperm1* (*NKD1*)	Zm00001d002654	INDETERMINATE DOMAIN transcription factor	aleurone-less	Yi et al. (2015)
	*Naked Endosperm1* (*NKD2*)	Zm00001d026113	INDETERMINATE DOMAIN transcription factor	aleurone-less	Yi et al. (2015)
	*Supernumerary Aleurone Layer1* (*SAL1*)	Zm00001d046599	Human Chmp1 homolog	multi-layered aleurone cells	Shen et al. (2003); Tian et al. (2007)
	*Thick Aleurone1* (*THK1*)	Zm00001d027278	NOT1 subunit of the CCR4-NOT complex	multi-layered aleurone cells	Yi et al. (2011)
	*ZmDof3*	Zm00001d035651	DNA binding with one finger (DOF) zinc-finger transcription factor	multi-layered aleurone cells	Qi et al. (2017)
	*Shrunken4* (*SH4*)	Zm00001d002797	YELLOW STRIPE-LIKE oligopeptide metal transporter ZmYSL2	lost aleurone cell characteristics	He et al. (2021)
	*Widow’s Peak Mutant1* (*WPK1*)	Zm00001d034383	Glutamate carboxypeptidase Viviparous8 (VP8)	suppressed anticlinal cell expansion of the aleurone	Suzuki et al. (2008)
	*Disorgal1* (*DIL1*)	not cloned	unknown	disorganized aleurone layer	Lid et al. (2004)
	*Disorgal2 (DIL2)*	not cloned	unknown	disorganized aleurone layer	Lid et al. (2004)
	*Extra Cell Layers1* (*XCL1*)	not cloned	unknown	abnormal aleurone cell mitotic division	Kessler et al. (2002)
	*Globby1* (*GLO1*)	not cloned	unknown	ectopically formed aleurone-like cells in the endosperm	Costa et al. (2003)

**Barley**	*Elongation2* (*ELO2*)	not cloned	unknown	abnormal aleurone cell size and shape, increased layer number	Lewis et al. (2009)
	*Defective Seed5*(*DES5*)	not cloned	unknown	reduced layer number of aleurone cells	Olsen et al. (2008)
**Rice**	*Thick Aleurone1* (*TA1*)	LOC_Os05g43440	mitochondrion-targeted single-stranded DNA-binding protein	increased number of aleurone cell layers	Li et al. (2021)
	*Thick Aleurone1* (*TA2*)	LOC_Os01g11900	DNA demethylase REPRESSOR OF SILENCING1 (OsROS1)	increased number of aleurone cell layers	Liu et al. (2018)
	*Fertilization-Independent Endosperm1* (*OsFIE1*)	LOC_Os08g04290	polycomb group protein	increased number of aleurone cell layers at the dorsal side of seed	Cheng et al. (2025)
	*RECA3*	LOC_Os01g67510	mitochondrial DNA recombinase	increased number of aleurone cell layers	Li et al. (2021)
	*TWINKLE*	LOC_Os06g45980	DNA helicase	increased number of aleurone cell layers	Li et al. (2021)
	*Rice prolamin box binding factor* (*RPBF*)	LOC_Os02g15350	DOF zinc-finger transcription factor	increased number of aleurone cell layers	Kawakatsu et al. (2009)
	*GRAIN WIDTH and WEIGHT2* (*GW2*)	LOC_Os02g14720	RING-type E3 ubiquitin ligase	increased aleurone cell size	Achary and Reddy (2021)
	*Protein Disulfide Isomerase Like 1-1* (*PDIL1-1*)	LOC_Os11g09280	protein disulfide isomerase	increased aleurone thickness	Kim et al. (2012)

Conversely, mutants such as *supernumerary aleurone layer1* (*sal1*) and *thick aleurone1* (*thk1*) in maize and *thick aleurone 1* (*ta1*) and *ta2* in rice develop multiple layers of aleurone in their seeds (Shen et al., 2003; Yi et al., 2011; Liu et al., 2018; Li et al., 2021), suggesting the presence of both positive and negative factors regulating aleurone differentiation (Figure 3B). Given that the *thk1*;*dek1* double mutant displays a phenotype similar to that of the *thk1* single mutant, *THK1* may act as a downstream factor of *DEK1* (Yi et al., 2011). The *thk1*;*nkd* mutant exhibits a greater number of aleurone layers than either single mutant, suggesting an additive effect between these genes in controlling aleurone cell differentiation (Yi et al., 2015). However, co-expression network analysis suggests that *THK1* and *NKD1/2* may epistatically regulate the same set of genes involved in cell cycle and division (Wu and Becraft, 2021).

Several genes that are not involved in aleurone cell fate determination are essential for aleurone cell division (Table 1). For example, the maize *disorgal1* (*dil1*) and *dil2* mutants display either relaxed or absent control over the mitotic division plane, resulting in mature grains with disorganized aleurone layers composed of irregularly shaped and sized cells (Lid et al., 2004). Similarly, the barley *elongation2* (*elo2*) mutant shows aleurone cell disorganization, with less regular cell sizes and shapes, and varying numbers ranging from one to six or seven (Lewis et al., 2009). A mutation in *Viviparous8* (*VP8*), which encodes a peptidase homologous to Arabidopsis ALTERED MERISTEM PROGRAM1, suppresses anticlinal cell expansion in maize aleurone, without affecting the periclinal plane (Suzuki et al., 2008). The maize *extra cell layers1* (*xcl1*) mutant displays periclinal divisions instead of the typical anticlinal divisions in the protodermal layer, resulting in an additional aleurone-like layer within the kernel (Kessler et al., 2002). The inner layer of these cells does not exhibit the starchy endosperm identities observed in the WT, indicating that *XCL1* plays a role in regulating both aleurone differentiation and cell division. The maize *glo1* mutant shows a range of aleurone defects, including the presence of multiple aleurone layers, irregular aleurone proliferation at the endosperm apex, and the absence of aleurone in certain regions (Costa et al., 2003). This suggests that the determination of aleurone cell fate and subsequent proliferation are two processes that are relatively independent, though some genes, such as *XCL1* and *GLO1*, may influence both events.

## Conservation of the molecular regulatory network in aleurone cell fate determination

**Figure 4:**
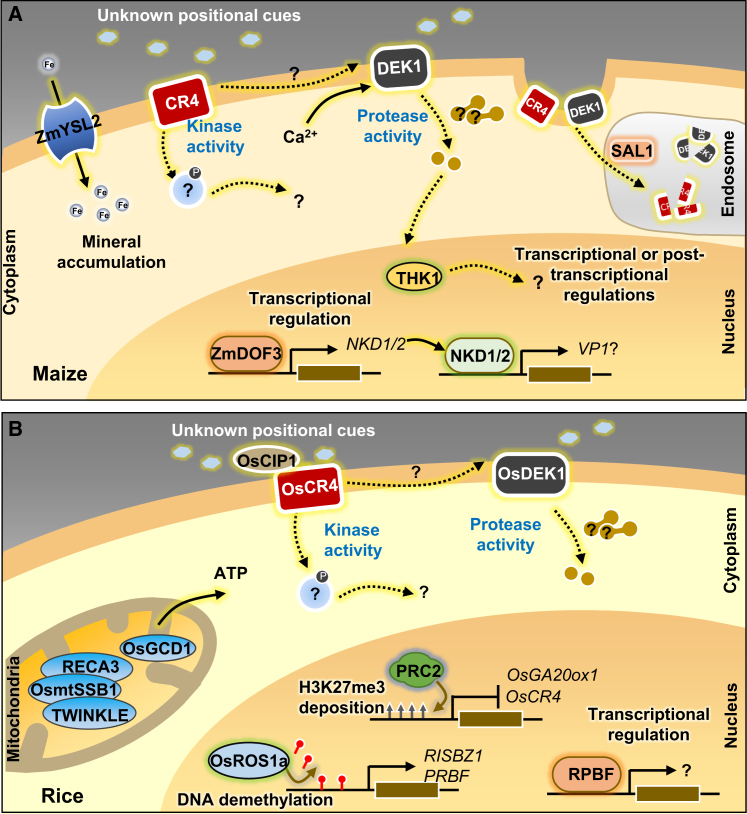
Molecular regulatory mechanisms of aleurone cell differentiation in maize and rice. **(A)** Maize CR4, a plasma membrane-localized receptor-like kinase, and DEK1, a plasma membrane-targeted protein with cytoplasmic calpain protease activity, potentially perceive positional cues released from endosperm cells to trigger downstream signaling. When activated, CR4 may phosphorylate an unidentified downstream target through its kinase activity to promote aleurone differentiation. Upon perception of the positional cue, the protease activity of DEK1 may be activated, leading to degradation of a yet-to-be-identified substrate to promote aleurone differentiation. The vacuolar sorting protein SAL1 regulates the concentration of DEK1 and CR4 in the plasma membrane through endosome-mediated degradation. THK1 acts downstream of DEK1 as a CCR4-NOT scaffold protein. ZmDOF3, a DOF family transcription factor, regulates the expression of *NKD1* and *NKD2*, which encode IDD family transcription factors that influence aleurone differentiation through transcriptional regulation. The YELLOW STRIPE-LIKE oligopeptide metal transporter ZmYSL2 is also involved in aleurone differentiation in maize. **(B)** OsCR4 and OsDEK1 play conserved roles in promoting aleurone differentiation in rice. The secreted protein OsCIP1 interacts with OsCR4 to stabilize it on the membrane. *OsCR4* expression is negatively regulated by the rice PRC2 complex through H3K27me3 modification. *OsGA20ox1,* another target of rice PRC2, positively regulates aleurone differentiation through GA biosynthesis in seeds. The DOF family transcription factor RPBF represses rice aleurone differentiation through transcriptional regulation. DNA at *RPBF* and *RISBZ1* loci is methylated, and the rice demethylase OsROS1a promotes the expression of these genes by removing methylation marks. In addition, the single-stranded DNA-binding protein OsmtSSB1 cooperates with the DNA recombinase RECA3 and the DNA helicase TWINKLE to repress aleurone differentiation in rice by maintaining the integrity of the mitochondrial genome. Similarly, the mitochondrion-localized factor OsGCD1 is involved in aleurone development in rice.

Over the past three decades, the molecular identities of several aleurone-related genes have been elucidated (Becraft et al., 1996; Lid et al., 2002; Shen et al., 2003; Gontarek et al., 2016; Liu et al., 2018; Wang et al., 2020; Li et al., 2021). These discoveries have significantly advanced our understanding of the mechanisms underlying aleurone cell fate determination and revealed the conservation of aleurone developmental regulatory networks across cereal plants (Figure 4A and 4B).

As a positive regulator essential for aleurone differentiation, CR4 is a proposed receptor-like kinase that perceives still-unidentified positional cues, thereby specifying aleurone cell fate (Becraft et al., 1996; Becraft and Asuncion-Crabb, 2000; Tian et al., 2007; Pu et al., 2012). The extracellular domain of rice CR4 (OsCR4) has been shown to interact with OsCR4 Interacting Protein1 (OsCIP1), a secreted protein localized to the outer surface of the plasma membrane (Yan et al., 2023). These interactions may contribute to the stabilization of OsCR4 on the membrane. Unlike *CR4*, which is expressed in all plant tissues, *OsCIP1* is exclusively expressed in the aleurone layer and pericarp of seeds (Pu et al., 2012; Wang et al., 2015). Notably, the loss-of-function mutant of *OsCIP1* exhibits a thinner aleurone layer, although aleurone differentiation is not disrupted (Wang et al., 2015; Yan et al., 2023), indicating that OsCIP1 itself does not determine cell fate.

DEK1, another key protein, targets the plasma membrane and comprises an extracellular loop located outside the cell and a cytoplasmic calpain protease (CALP) domain situated inside the cell (Lid et al., 2002; Tian et al., 2007). Over the past two decades, studies have shown that DEK1 is one of a few key genes for the evolution of meristems in land plants, although its biochemical function remains elusive (Olsen et al., 2015). In *Arabidopsis, t*he CALP domain of DEK1 can be released from the plasma membrane through autolytic cleavage (Johnson et al., 2008) and its protease activity can be enhanced by calcium ions (Wang et al., 2003). The CALP domain has been shown to function as an effector component in the process of mechanotransduction (Tran et al., 2017). Despite the ability of the CALP domain to mitigate seed abortion phenotypes in the *Arabidopsis dek1* mutant, the resulting seeds still fail to develop an aleurone layer (Tian et al., 2007),suggesting that the extracellular loop of DEK1 is indispensable for aleurone cell fate determination. A recent study suggests that the DEK1 protease marks proteins for degradation via the N-end rule degradation pathway (Demko et al., 2024). It is inferred that DEK1 may interact with extracellular positional cues and other ligands to activate the intracellular calpain protease activity, thereby transmitting signals for cell fate programming (Becraft and Yi, 2011). However, another study has proposed that the loop of DEK1 may be an intracellular domain (Kumar et al., 2010). If this hypothesis is true, the current model needs to be reevaluated. DEK1 and CR4 are localized in the endosome and show a pattern of colocalization with the class E vacuolar sorting protein SAL1 (Shen et al., 2003; Tian et al., 2007). As a negative regulator of aleurone cell fate determination, SAL1 may control the concentration of DEK1 and CR4 in the plasma membrane through an endosome-mediated degradation pathway (Tian et al., 2007).

Transcriptional regulation is a critical determinant of aleurone cell fate. For example, aleurone defects observed in the maize *nkd* mutant are caused by the actions of *NKD1* and *NKD2*, two duplicated genes encoding INDETERMINATE DOMAIN (IDD) TFs. In developing maize endosperm, NKDs transcriptionally activate genes such as *Opaque2* and *Viviparous1* (*VP1*). The *VP1* gene is exclusively expressed in the aleurone layer and the embryo of both maize and rice (Miyoshi et al., 2002; Cao et al., 2007; Zheng et al., 2019), suggesting a possible role of VP1 in aleurone development. *NKDs* are downstream targets of the DNA Binding with One Finger (DOF) family of TFs. The expression of *NKD1* and *NKD2* was found to be significantly reduced in maize *ZmDof3* knockdown lines (Qi et al., 2017). Additionally, the knockdown of *Rice Prolamin Box Binding Factor* (*RPBF*), a *ZmDof3* homolog in rice, resulted in the development of multi-layered aleurone cells in the seed. This phenotype can be enhanced by simultaneously knocking down the bZIP TF gene *Rice Basic Leucine Zipper1* (*RISBZ1*), although suppression of *RISBZ1* alone does not affect aleurone differentiation in rice (Kawakatsu et al., 2009). The expression of *OsDEK1*, *OsCR4*, and *OsSAL1* was significantly repressed in either the *RPBF* single mutant or *RPBF* and *RISBZ1* double knockdown mutants, indicating that these aleurone determinants are potential downstream targets of these TFs (Kawakatsu et al., 2009). In rice, inactivation of positive regulators of aleurone differentiation, such as *OsDEK1*, *OsCR4*, and *OsSAL1*, in the multi-layered aleurone cells suggests the presence of a feedback loop for self-regulation of aleurone differentiation. THK1 encodes a homolog of NEGATIVE ON TATA-LESS1 (NOT1), a protein that acts as a scaffold for the CARBON CATABOLITE REPRESSION4-NEGATIVE ON TATA-LESS (CCR4-NOT) complex (Wu et al., 2020). CCR4-NOT is a conserved eukaryotic protein complex that regulates gene expression at multiple levels, from the production of messenger RNAs in the nucleus to their degradation in the cytoplasm (Collart, 2016). Therefore, THK1 may regulate aleurone differentiation at either the transcriptional or post-transcriptional level.

Epigenetic modification provides an additional regulatory layer for aleurone development. It has been demonstrated that epigenetic marks, such as DNA methylation and histone modification, undergo dynamic reprogramming during seed development (Kawashima and Berger, 2014). The REPRESSOR OF SILENCING 1 (ROS1)/DEMETER (DME) family of DNA demethylases plays a role in DNA demethylation in all eukaryotes (Zhang et al., 2018). Null mutations of rice *ROS1a* (*OsROS1a*) result in a range of reproductive defects, including failures in gametogenesis, endosperm abortion, and embryogenesis abnormalities (Ono et al., 2012; Zhou et al., 2021). However, rice *ta2* mutants with weak *osros1a* alleles produce viable seeds with multi-layered aleurone (Liu et al., 2018), implicating that increased DNA methylation at the promoter regions of *RPBF* and *RISBZ1* leads to their downregulation and promotes aleurone differentiation (Liu et al., 2018). A recent study demonstrated that the defect in rice Fertilization Independent Endosperm1 (OsFIE1) may also induce aleurone thickening at the dorsal side of the seed, resulting from a reduction in tri-methylated histone H3 at lysine 27 (H3K27me3) modifications (Cheng et al., 2025). Specifically, H3K27me3 marks are completely depleted at the *OsCR4* locus, associated with the gene’s upregulation in the aleurone cells of *osfie1* and other mutants of the Polycomb Repressive Complex 2 (PRC2) members (Cheng et al., 2021, 2025). This indicates that PRC2-mediated H3K27me3 modification is crucial for aleurone development in rice. Moreover, multiple genes involved in GA biosynthesis, including the rice *GA 20-oxidase1* (*OsGA20ox1*), exhibited reduced levels of H3K27me3, resulting in their activation in *osfie1* (Cheng et al., 2025). The number of aleurone layers in the *osfie1;osga20ox1* double mutant was significantly reduced compared with that of the WT, suggesting that PRC2 may modulate aleurone differentiation by influencing GA biosynthesis in rice.

Rice *TA1*, designated OsmtSSB1, encodes a mitochondrion-targeted protein with single-stranded DNA-binding activity (Li et al., 2021). Defects in OsmtSSB1-interacting mitochondrial DNA recombinase RECA3 and DNA helicase TWINKLE result in a *ta1*-like phenotype characterized by an increased number of subaleurone cell layers (Li et al., 2021). Another mitochondrion-localized factor involved in aleurone development is Rice GAMETE CELLS DEFECTIVE1 (OsGCD1). The *osgcd1* mutant exhibits delayed and aberrant differentiation of aleurone cells (Huang et al., 2017); although the aleurone layer is present at the mature stage, the cells are enlarged and disorganized, with significant variation in the number of cell layers (Huang et al., 2017). These findings indicate that mitochondrial development plays a pivotal role in determining the fate of aleurone cells.

Furthermore, the *GRAIN WIDTH and WEIGHT2* (*GW2*) gene, which encodes a RING-type E3 ubiquitin ligase, and the *PDIL1-1* gene, which encodes a protein disulfide isomerase assisting in protein folding, may influence aleurone development in rice. This is based on the increased aleurone thickness in the *gw2* and *pdil1-1* mutants (Kim et al., 2012; Achary and Reddy, 2021). However, the precise mechanisms remain elusive.

Phenotypic parallels observed in some regulators involved in aleurone development reveal the conservation of the regulatory networks across cereal plants. For instance, partial absence of the aleurone layer in maize *dek1* and *cr4* loss-of-function mutants is observed in severe rice *ADAXIALIZED LEAF1* (the rice homolog of *DEK1*) mutants and *OsCR4* knockdown rice lines (Hibara et al., 2009; Pu et al., 2012). Furthermore, defects in DOF family TFs, namely the maize ZmDOF3 and rice RPBF, result in a comparable multi-layered aleurone phenotype (Kawakatsu et al., 2009; Qi et al., 2017). These observations suggest the presence of conserved regulatory mechanisms of aleurone identity determination across cereals. This assertion is further substantiated by the disrupted expression of *CR4*, *DEK1*, and *SAL1* in the rice *RPBF* knockdown and barley *des5* null mutants, both of which exhibit defective aleurone development (Olsen et al., 2008; Kawakatsu et al., 2009).

## Hormone regulation and aleurone development

Phytohormones play a critical role in the regulation of seed development (Zhang et al., 2021), influencing processes such as aleurone differentiation, maturation, and programmed cell death during germination (Becraft and Yi, 2011). However, the specific mechanisms underlying their action remain to be fully elucidated.

Evidence suggests a potential involvement of cytokinin and auxin in aleurone cell differentiation. For example, expression of the cytokinin-synthesizing gene *isopentenyl transferase* (*IPT*) in maize, driven by an *Arabidopsis* senescence-inducible promoter from the Cys protease gene *SAG12*, results in interspersed patches of aleurone and starchy endosperm cells at the periphery of the crown region of kernels (Geisler-Lee and Gallie, 2005). These results suggest that cytokinin may inhibit aleurone differentiation. Conversely, the application of *N*-1-naphthylphthalamic acid, an inhibitor of auxin transporters, induces the formation of a multi-layered aleurone in maize (Forestan et al., 2010), suggesting that auxin may positively influence aleurone fate determination. Furthermore, significant upregulation of numerous auxin-responsive genes is observed in the kernels of maize *nkd1,2* double mutants, which exhibit increased auxin concentrations in the outermost cell layers (Wu and Becraft, 2021). In addition, the exclusive expression of specific auxin biosynthetic and signaling genes (e.g., rice *YUCCA12* and rice *indole-3-acetic acid inducible29*) within the aleurone layer suggests a role for auxin in aleurone development (Basunia et al., 2021). However, seed-specific *YUCCA* (*YUC*) genes, including maize *YUC1* and rice *YUC11*, appear to be unrelated to aleurone development. This is evidenced by the normal development of aleurone in mutant plants exhibiting severe kernel defects (Bernardi et al., 2012; Xu et al., 2021), highlighting the complexity of aleurone differentiation and development regulation by auxin. The underlying mechanisms are a subject of considerable interest and warrant further exploration.

Defects in maize VP8 have been shown to reduce ABA levels and suppress the anticlinal expansion of aleurone cells (Suzuki et al., 2008). However, the fact that other ABA-deficient mutants do not exhibit a similar phenotype calls into question the significance of ABA in aleurone development (Becraft and Yi, 2011). It is well established that GA is necessary to induce vacuolation of aleurone cells during germination (Bethke et al., 2007; Zhang et al., 2020). A recent study reported that rice null *osfie1* mutants produced significantly more layers of aleurone cells, with extremely large vacuoles observed within these cells (Cheng et al., 2025). This phenomenon is presumably due to the overaccumulation of GA, which triggers aleurone vacuolation in *osfie1* seeds. The application of GA intensified the multi-layered aleurone phenotype of *osfie1*, whereas the knockout of *OsGA20ox1*, a GA biosynthetic gene predominantly expressed in the developing caryopsis of rice, in the *osfie1* background successfully reduced the number of aleurone layers (Cheng et al., 2025). These findings suggest that GA is required for inducing aleurone differentiation. However, the study also demonstrated that the *OsGA20ox1* defect in the WT background had no impact on aleurone thickening. Similarly, exogenous GA treatment exhibited a limited effect on WT aleurone thickness. These results imply that while GA is necessary, it is not sufficient to promote aleurone differentiation in rice. Further investigation is required to elucidate the underlying mechanisms.

## Potential utilization of aleurone-related traits

**Figure 5:**
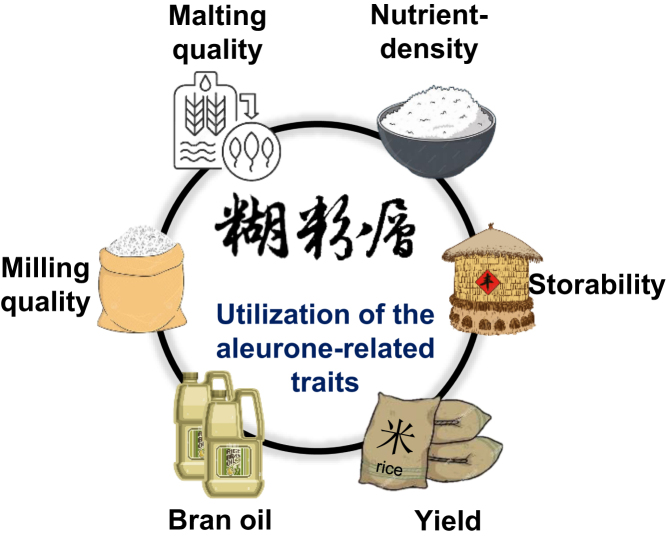
Utilization of the aleurone-related traits. Aleurone-related traits have potential to be widely utilized for enhancing the nutritional value, milling and malting characteristics, seed storability, and yield-related traits of cereal crops. In addition, the aleurone can be used to enhance the production of bran oil, a valuable source of nutritionally beneficial compounds.

The study of aleurone has a long and distinguished history in the scientific community. The accumulation of anthocyanins in maize aleurone cells provides a unique genetic system for investigating the inheritance behavior of genes (Candela and Hake, 2008). The aleurone system has been instrumental in the discovery of several breakthrough concepts, such as transposable elements, gene imprinting, and paramutation (McClintock, 1950; Brink, 1956; Kermicle, 1970). Additionally, the cereal aleurone serves as an excellent model for studying hormonal signaling in plants (Bethke et al., 1997). Recently, interest in aleurone research has significantly increased due to its biological significance and potential health benefits (Figure 5) (Atwell et al., 2007; Brouns et al., 2012; Meziani et al., 2021; Lebert et al., 2022).

From a practical standpoint, cereals with a thicker aleurone layer offer advantages for consumers of whole grains due to the increased nutrient density in the seed. For example, maize landraces with the MAL trait have shown elevated contents of total protein, amino acids, anthocyanins, iron, and zinc, along with increased aleurone cell layers in the kernel (Wolf et al., 1972; Nelson and Chang, 1974; Paulsmeyer and Juvik, 2023). Maize *sugary1* (*su1*) kernels contain approximately twice the niacin content compared to normal starchy *Su1* kernels, attributed to a greater quantity of aleurone tissue (Teas, 1952). In rice, the *ta1* allele was successfully introduced into the variety Zixiangnuo 1306 by backcrossing, resulting in the black rice line Zhongzi-1, which exhibits elevated levels of nutritional factors such as proteins, lipids, dietary fibers, vitamins, and minerals without any yield penalty compared to the recurrent parent (Li et al., 2021). Barley, which is significant for the malting and brewing industries, benefits from its multi-layered aleurone, which produces a greater amount of amylase. This enzyme catalyzes the conversion of starch into fermentable sugars during the malting process (Aubert et al., 2018). Enhancing the number or thickness of the aleurone layers could improve the malting quality of barley and extend the use of cereal crops, such as maize varieties with the MAL trait, for similar malting purposes.

The typical diet in most regions primarily consists of highly refined grains that have been stripped of their outer bran and milled into a fine-textured carbohydrate. Reducing the number of aleurone cells in seeds may increase the rate of starchy endosperm production. Aleurone is predicted to account for 6.5% of the weight of the wheat grain (Barron et al., 2007). According to the United Nations' Food and Agriculture Organization (FAO), global wheat production in 2022 reached a record 794 million tons (www.fao.org). Planting aleurone-less wheat could result in approximately 50 million tons of additional carbohydrate production, provided there is no yield penalty for aleurone-less grains. Although challenging to attain, the potential benefits of this approach warrant further consideration. Furthermore, the predominant accumulation of lipids in the aleurone suggests that grains with no or fewer aleurone cells may have an extended shelf life, given the negative association between seed lipid content and storability (Zhou et al., 2024). Therefore, a thin aleurone may also be beneficial for the improvement of cereals, depending on the objectives of the breeding program and the intended end use of the grains.

The aleurone layer constitutes approximately 50%–70% of the bran weight of wheat (Pascoe and Fulcher, 2007), representing the primary source of bioactive nutrients accumulated in the bran tissues. Consequently, in addition to its use in the animal feed industry, the bran is attracting greater interest for the production of biofortified foods and bran oil (Gul et al., 2015; Bagdi et al., 2016). Enhancements of aleurone-related characteristics may also prove advantageous for the expanded industrial utilization of the bran. For example, white flour products of aleurone-biofortified wheat exhibit significantly reduced bitterness, firmness, and dark coloration compared with whole grain flour, while maintaining a comparable nutritional composition (Bagdi et al., 2016; Li et al., 2019; Zhou et al., 2022).

## Challenges and potential solutions

The primary challenge in improving aleurone-related traits is phenotyping. Although new approaches for genetic analysis of these traits have been developed (Kim et al., 2022; Nguyen et al., 2022; Xu et al., 2023b), they typically involve time-consuming sectioning and staining. Three-dimensional and non-destructive technologies, such as X-ray computed tomography and fast X-ray fluorescence microscopy, have proven effective in determining aleurone-related traits with high resolution (Chen et al., 2021; Legland et al., 2022; Ren et al., 2023). However, these methods require expensive equipment, and their low throughput limits their use in large-scale screening in breeding. Near-infrared reflectance spectroscopy offers a rapid, non-destructive method and has demonstrated its capacity to predict pericarp thickness in whole sorghum grains (Guindo et al., 2016), indicating its potential for phenotyping aleurone-related traits.

Identifying aleurone mutants in species such as rice and wheat remains challenging, hindering our understanding of the regulatory networks governing aleurone development and obstructing the enhancement of aleurone-related traits through contemporary breeding techniques, including transgenic breeding, molecular design, and gene editing. Combining traditional and targeted mutagenesis of seed-preferential genes (Zhao et al., 2024) with the aforementioned novel phenotyping technologies may facilitate the identification of new aleurone regulators in plants. Similar to maize, several barley varieties exhibit a blue aleurone layer due to anthocyanin accumulation (Xu et al., 2023a). This phenotype is a valuable yet often overlooked feature that could aid in isolating new aleurone regulators in barley.

**Figure 6:**
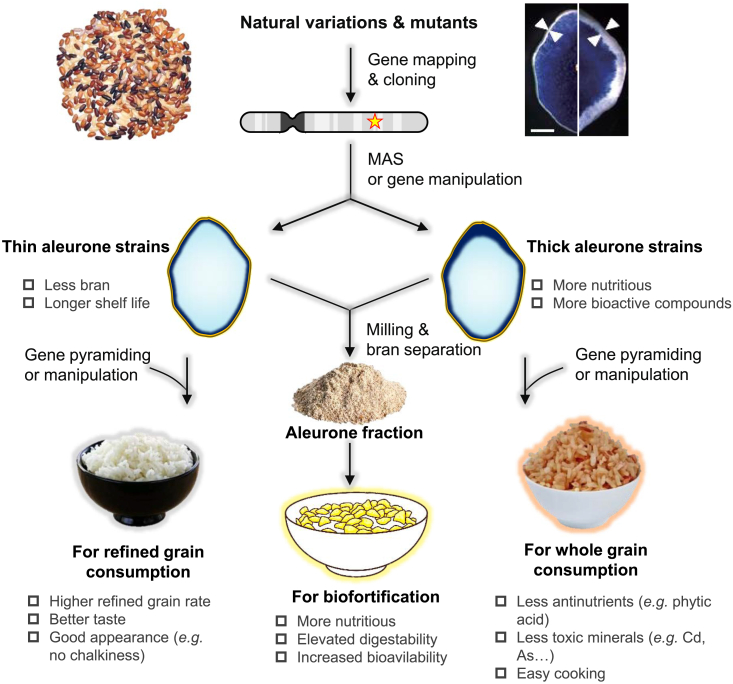
Strategies to enhance aleurone-related traits in cereals. Implementing high-throughput phenotyping will streamline the process for screening varieties or mutants with specific aleurone characteristics. The underlying causal genes can be cloned using linkage or association populations. Subsequently, gene editing, transgenic breeding, or traditional gene pyramiding strategies may be employed to facilitate the improvement of aleurone-related traits. Traits such as reduced aleurone cells or increased aleurone cells can be leveraged for crop improvement; the former may enhance shelf life and milling yield, while the latter may increase the nutritional compounds in grains. These traits may be combined with improved appearance, taste, and cooking qualities through traditional or modern breeding technologies, including marker-assisted selection, gene editing, and transgenic breeding. Moreover, enhancing the efficiency of aleurone cell isolation from bran could be beneficial for food biofortification efforts that utilize these isolated aleurone cells.

Most known aleurone regulators have proven ineffective for improving aleurone-related traits due to undesirable phenotypes, such as growth retardation, defective embryos, and floury endosperm, caused by the pleiotropic effects of these genes (Becraft et al., 1996; Becraft and Asuncion-Crabb, 2000; Shen et al., 2003; Kawakatsu et al., 2009; Li et al., 2021). The constitutive expression profiles of these genes are thought to contribute to these unfavorable traits that are coupled with the desired aleurone traits. A promising approach is to manipulate gene expression exclusively in aleurone cells using aleurone-specific promoters or, alternatively, edit promoters or *cis*-elements using clustered regularly interspaced short palindromic repeats (CRISPR) technology to modify spatio-temporal gene expression (Li et al., 2024). *Geng* rice varieties typically exhibit thicker aleurone than *Xian* varieties (Hoshikawa, 1967; Khin et al., 2013, She et al., 2025), yet both subspecies display robust vigor. Similarly, no deleterious effects were identified in maize landraces with multi-layered aleurone (Paulsmeyer and Juvik, 2023). These findings indicate that aleurone traits can be decoupled from the unfavorable traits seen in existing single-gene mutants. Previous studies have demonstrated that aleurone thickness is a highly heritable trait in cereals, with an estimated broad heritability of approximately 60% in rice and maize (Jestin et al., 2008; Xu et al., 2023b; Paulsmeyer and Juvik, 2023). It is therefore optimal to leverage natural variations to breed new varieties with favorable aleurone-related traits. However, given the wide range of phenotypic variation observed even within a homogeneous line, precise phenotyping is crucial for the effectiveness of selection. Identifying and cloning QTLs responsible for aleurone development using either diversity panels (for association-based analysis) or bi-parental populations (for linkage-based analysis) is a crucial step in the breeding process (Figure 6). Marker-assisted selection can significantly enhance the effectiveness of the selection process while reducing labor inputs. Despite the limitations of current phenotyping technology, few studies have attempted to investigate the genetic basis of aleurone-related traits (Jestin et al., 2008; Khin et al., 2012; Kim et al., 2022; Xu et al., 2023b; Paulsmeyer and Juvik, 2023). However, several studies have demonstrated the existence of stable loci consistently expressed across diverse environments (Xu et al., 2023b), providing a promising foundation for strategies that utilize natural variation to enhance aleurone-related traits.

Recent advancements in spatial transcriptomics and single-cell transcriptomics have facilitated the development of high-resolution expression maps for developing grains in rice and maize (Fu et al., 2023; Zhou et al., 2023; Yuan et al., 2024). These maps offer a valuable resource for understanding the molecular regulatory mechanisms of aleurone differentiation and development. Numerous genes have been identified as either exclusively or predominantly expressed in the aleurone, or as being activated during aleurone cell differentiation. Understanding the biological significance of these factors for aleurone development holds great potential for advancing research in this field and identifying potential genetic resources for enhancing aleurone-related traits.

In addition to essential minerals like calcium, iron, zinc, magnesium, and potassium, the aleurone layer also contains high levels of toxic elements such as arsenic and cadmium (Wei et al., 2017; Jo and Todorov, 2019), posing potential health risks associated with consuming whole grain cereals with enhanced aleurone. Antinutrients like phytic acid, which binds minerals and prevents their absorption, are also concentrated in the aleurone layer (Iwai et al., 2012). Breeders must address these issues associated with a multi-layered aleurone. A recent study demonstrated that increasing aleurone thickness significantly elevates the zinc content in rice while reducing the accumulation of toxic cadmium (She et al., 2025), suggesting that enhancing aleurone does not necessarily lead to the over-accumulation of toxic minerals. Some cereal varieties exhibit markedly lower concentrations of toxic minerals or phytic acid in their seeds. Modifying the aleurone trait in these varieties background may be an effective strategy for mitigating the adverse effects of a thick aleurone. Pyramiding optimal genes/alleles through traditional breeding or the integration of genetic and genomic technologies could produce healthier cereals for human consumption (Figure 6).

Finally, it is crucial to note that in cereals, most breeding strategies using aleurone-related traits have not undergone rigorous evaluation and require further investigation. The seed is a highly sophisticated organ that integrates signals from maternal tissues as well as the filial endosperm and embryo to regulate development (Doll and Ingram, 2022). The impact of manipulating aleurone traits on seed development and yield remains unclear. For example, aleurone cells are indispensable in seed germination (Penfield et al., 2004; Hong et al., 2012), leading to the reasonable hypothesis that seeds lacking the aleurone layer may display germination defects. Moreover, while seeds with a thickened aleurone may offer enhanced nutritional qualities, it is important to consider potential trade-offs in terms of appearance, milling, or cooking qualities. How might the linkage of targeted aleurone traits and undesirable traits be uncoupled? Answering these questions will establish a solid foundation for fruitful research and ultimately benefit the breeding of cereals with enhanced aleurone-related traits.

## Funding

This research was supported by grants from the Biological Breeding-National Science and Technology Major Project (2023ZD0406802), the 10.13039/501100001809National Natural Science Foundation of China (32170344), the Jiangsu Agricultural Science and Technology Innovation Fund (CX(23)3096), and the project funded by the 10.13039/501100012246Priority Academic Program Development of Jiangsu Higher Education Institutions (PAPD).

## Acknowledgments

No conflict of interest declared.

## Author contributions

H.L., J.Z., and C.C. performed the literature search, and H.L. and C.C. wrote the paper.

## References

[bib1] Achary V.M.M., Reddy M.K. (2021). CRISPR-Cas9 mediated mutation in GRAIN WIDTH and WEIGHT2 (GW2) locus improves aleurone layer and grain nutritional quality in rice. Sci. Rep..

[bib2] Ahibuya T. (1929). On the Thickness of Aleurone Layer in Rice Kernel. J. Soc. Trop. Agric..

[bib3] Aslam M.F., Ellis P.R., Berry S.E., Latunde-Dada G.O., Sharp P.A. (2018). Enhancing mineral bioavailability from cereals: Current strategies and future perspectives. Nutr. Bull..

[bib4] Atwell B., von Reding W., Earling J., Kanter M., Snow K. (2007). Whole Grains and Health.

[bib5] Aubert M.K., Coventry S., Shirley N.J., Betts N.S., Würschum T., Burton R.A., Tucker M.R. (2018). Differences in hydrolytic enzyme activity accompany natural variation in mature aleurone morphology in barley (Hordeum vulgare L.). Sci. Rep..

[bib6] Bagdi A., Tóth B., Lőrincz R., Szendi S., Gere A., Kókai Z., Sipos L., Tömösközi S. (2016). Effect of aleurone-rich flour on composition, baking, textural, and sensory properties of bread. Lwt.

[bib7] Barron C., Surget A., Rouau X. (2007). Relative amounts of tissues in mature wheat (Triticum aestivum L.) grain and their carbohydrate and phenolic acid composition. J. Cereal. Sci..

[bib8] Basunia M.A., Nonhebel H.M., Backhouse D., McMillan M. (2021). Localised expression of OsIAA29 suggests a key role for auxin in regulating development of the dorsal aleurone of early rice grains. Planta.

[bib9] Becraft P.W., Asuncion-Crabb Y. (2000). Positional cues specify and maintain aleurone cell fate in maize endosperm development. Development.

[bib10] Becraft P.W., Yi G. (2011). Regulation of aleurone development in cereal grains. J. Exp. Bot..

[bib11] Becraft P.W., Stinard P.S., McCarty D.R. (1996). CRINKLY4: A TNFR-like receptor kinase involved in maize epidermal differentiation. Science.

[bib12] Becraft P.W., Li K., Dey N., Asuncion-Crabb Y. (2002). The maize dek1 gene functions in embryonic pattern formation and cell fate specification. Development.

[bib13] Bernardi J., Lanubile A., Li Q.-B., Kumar D., Kladnik A., Cook S.D., Ross J.J., Marocco A., Chourey P.S. (2012). Impaired auxin biosynthesis in the defective endosperm18 mutant is due to mutational loss of expression in the ZmYuc1 gene encoding endosperm-specific YUCCA1 protein in maize. Plant Physiol..

[bib14] Bethke P.C., Schuurink R., Jones R.L. (1997). Hormonal signalling in cereal aleurone. J. Exp. Bot..

[bib15] Bethke P.C., Libourel I.G.L., Aoyama N., Chung Y.Y., Still D.W., Jones R.L. (2007). The arabidopsis aleurone layer responds to nitric oxide, gibberellin, and abscisic acid and is sufficient and necessary for seed dormancy. Plant Physiol..

[bib16] Brink R.A. (1956). a Genetic Change Associated With the R Locus in Maize Which Is Directed and Potentially Reversible. Genetics.

[bib17] Brouns F., Hemery Y., Price R., Anson N.M. (2012). Wheat Aleurone: Separation, Composition, Health Aspects, and Potential Food Use. Crit. Rev. Food Sci. Nutr..

[bib18] Candela H., Hake S. (2008). The art and design of genetic screens: Maize. Nat. Rev. Genet..

[bib19] Cao X., Costa L.M., Biderre-Petit C., Kbhaya B., Dey N., Perez P., McCarty D.R., Gutierrez-Marcos J.F., Becraft P.W. (2007). Abscisic acid and stress signals induce Viviparous1 expression in seed and vegetative tissues of maize. Plant Physiol..

[bib20] Casacuberta J.M., Puigdomènech P., San Segundo B. (1991). A gene coding for a basic pathogenesis-related (PR-like) protein from Zea mays. Molecular cloning and induction by a fungus (Fusarium moniliforme) in germinating maize seeds. Plant Mol. Biol..

[bib21] Charlton W.L., Keen C.L., Merriman C., Lynch P., Greenland A.J., Dickinson H.G. (1995). Endosperm development in Zea mays; implication of gametic imprinting and paternal excess in regulation of transfer layer development. Development.

[bib22] Chen K., Zhang W., La T., Bastians P.A., Guo T., Cao C. (2021). Microstructure investigation of plant architecture with X-ray microscopy. Plant Sci..

[bib23] Chen Z., Mense A.L., Brewer L.R., Shi Y.-C. (2024). Wheat bran layers: composition, structure, fractionation, and potential uses in foods. Crit. Rev. Food Sci. Nutr..

[bib24] Cheng X., Pan M., Zhiguo E., Zhou Y., Niu B., Chen C. (2021). The maternally expressed polycomb group gene OsEMF2a is essential for endosperm cellularization and imprinting in rice. Plant Commun..

[bib25] Cheng X., Zhang S.E.Z., Yang Z., Cao S., Zhang R., Niu B., Li Q.-F., Zhou Y., Huang X.Y. (2025). Maternally expressed FERTILIZATION-INDEPENDENT ENDOSPERM1 regulates seed dormancy and aleurone development in rice. Plant Cell.

[bib26] Cho J. (1956). Double Fertilization in Oryza sativa L and Development of the Endosperm with Special Reference to the Aleurone Layer. Bull. Natl. Inst. Agric. Sci. Ser. D (Physiol. Genet.).

[bib27] Collart M.A. (2016). The Ccr4-Not complex is a key regulator of eukaryotic gene expression. Wiley Interdiscip. Rev. RNA.

[bib28] Costa L.M., Gutierrez-Marcos J.F., Brutnell T.P., Greenland A.J., Dickinson H.G. (2003). The globby1-1 (glo1-1) mutation disrupts nuclear and cell division in the developing maize seed causing alterations in endosperm cell fate and tissue differentiation. Development.

[bib29] Demko V., Belova T., Messerer M., Hvidsten T.R., Perroud P.F., Ako A.E., Johansen W., Mayer K.F.X., Olsen O.A., Lang D. (2024). Regulation of developmental gatekeeping and cell fate transition by the calpain protease DEK1 in Physcomitrium patens. Commun. Biol..

[bib30] Doll N.M., Ingram G.C. (2022). Embryo–Endosperm Interactions. Annu. Rev. Plant Biol..

[bib31] Drea S., Leader D.J., Arnold B.C., Shaw P., Dolan L., Doonan J.H. (2005). Systematic spatial analysis of gene expression during wheat caryopsis development. Plant Cell.

[bib32] Duangploy S., Zuber M.S., Cumbie B. (1976). Inheritance of multiple aleurone layering. Maize Genet. Coop. Newsl..

[bib33] Forestan C., Meda S., Varotto S. (2010). ZmPIN1-Mediated Auxin Transport Is Related to Cellular Differentiation during Maize Embryogenesis and Endosperm Development. Plant Physiol..

[bib34] Fu Y., Xiao W., Tian L., Guo L., Ma G., Ji C., Huang Y., Wang H., Wu X., Yang T. (2023). Spatial transcriptomics uncover sucrose post-phloem transport during maize kernel development. Nat. Commun..

[bib35] Geisler-Lee J., Gallie D.R. (2005). Aleurone cell identity is suppressed following connation in maize kernels. Plant Physiol..

[bib36] Gontarek B.C., Becraft P.W., Larkins B.A. (2017). Maize Kernel Development.

[bib37] Gontarek B.C., Neelakandan A.K., Wu H., Becraft P.W. (2016). NKD transcription factors are central regulators of maize endosperm development. Plant Cell.

[bib38] Gruis D.F., Guo H., Selinger D., Tian Q., Olsen O.A. (2006). Surface position, not signaling from surrounding maternal tissues, specifies aleurone epidermal cell fate in maize. Plant Physiol..

[bib39] Guindo D., Davrieux F., Teme N., Vaksmann M., Doumbia M., Fliedel G., Bastianelli D., Verdeil J.L., Mestres C., Kouressy M. (2016). Pericarp thickness of sorghum whole grain is accurately predicted by NIRS and can affect the prediction of other grain quality parameters. J. Cereal. Sci..

[bib40] Gul K., Yousuf B., Singh A.K., Singh P., Wani A.A. (2015). Rice bran: Nutritional values and its emerging potential for development of functional food - A review. Bioact. Carbohydrates Diet. Fibre.

[bib41] Guo H., Wu H., Sajid A., Li Z. (2022). Whole grain cereals: the potential roles of functional components in human health. Crit. Rev. Food Sci. Nutr..

[bib42] Hands P., Drea S. (2012). A comparative view of grain development in Brachypodium distachyon. J. Cereal. Sci..

[bib43] He Y., Yang Q., Yang J., Wang Y.-F., Sun X., Wang S., Qi W., Ma Z., Song R. (2021). shrunken4 is a mutant allele of ZmYSL2 that affects aleurone development and starch synthesis in maize. Genetics.

[bib44] Hermans W., Mutlu S., Michalski A., Langenaeken N.A., Courtin C.M. (2021). The Contribution of Sub-Aleurone Cells to Wheat Endosperm Protein Content and Gradient Is Dependent on Cultivar and N-Fertilization Level. J. Agric. Food Chem..

[bib45] Hibara K.I., Obara M., Hayashida E., Abe M., Ishimaru T., Satoh H., Itoh J.I., Nagato Y. (2009). The ADAXIALIZED LEAF1 gene functions in leaf and embryonic pattern formation in rice. Dev. Biol..

[bib46] Hong Y.-F., Ho T.-H.D., Wu C.-F., Ho S.-L., Yeh R.-H., Lu C.-A., Chen P.-W., Yu L.-C., Chao A., Yu S.-M. (2012). Convergent starvation signals and hormone crosstalk in regulating nutrient mobilization upon germination in cereals. Plant Cell.

[bib47] Hong S., Go J., Kim J.-H., Jo J., Kim J.W., Park J.S., Ro N., Yi G. (2024). A core collection enriched for Korean maize (Zea mays L.) landraces having kernel texture related morphological characters and novel multi-aleurone layer phenotypes. J. Agric. Food Res..

[bib48] Hoshikawa K. (1967). Studies on the Development of Endosperm in Rice : 5. The number of aleuron cell layers, its varietal difference and the influence of environmental factors. Jpn. J. Crop Sci..

[bib49] Huang X., Peng X., Sun M.X. (2017). OsGCD1 is essential for rice fertility and required for embryo dorsal-ventral pattern formation and endosperm development. New Phytol..

[bib50] Iwai T., Takahashi M., Oda K., Terada Y., Yoshida K.T. (2012). Dynamic changes in the distribution of minerals in relation to phytic acid accumulation during rice seed development. Plant Physiol..

[bib51] Jääskeläinen A.S., Holopainen-Mantila U., Tamminen T., Vuorinen T. (2013). Endosperm and aleurone cell structure in barley and wheat as studied by optical and Raman microscopy. J. Cereal. Sci..

[bib52] Jestin L., Ravel C., Auroy S., Laubin B., Perretant M.R., Pont C., Charmet G. (2008). Inheritance of the number and thickness of cell layers in barley aleurone tissue (Hordeum vulgare L.): An approach using F2-F3 progeny. Theor. Appl. Genet..

[bib53] Jo G., Todorov T.I. (2019). Distribution of nutrient and toxic elements in brown and polished rice. Food Chem..

[bib54] Johnson K.L., Faulkner C., Jeffree C.E., Ingram G.C. (2008). The phytocalpain defective kernel 1 is a novel Arabidopsis growth regulator whose activity is regulated by proteolytic processing. Plant Cell.

[bib55] Kawakatsu T., Yamamoto M.P., Touno S.M., Yasuda H., Takaiwa F. (2009). Compensation and interaction between RISBZ1 and RPBF during grain filling in rice. Plant J..

[bib56] Kawashima T., Berger F. (2014). Epigenetic reprogramming in plant sexual reproduction. Nat. Rev. Genet..

[bib57] Kermicle J.L. (1970). Dependence of the R-mottled aleurone phenotype in maize on mode of sexual transmission. Genetics.

[bib58] Kessler S., Seiki S., Sinha N. (2002). Xcl1 causes delayed oblique periclinal cell divisions in developing maize leaves, leading to cellular differentiation by lineage instead of position. Development.

[bib59] Khin O.M., Matsue Y., Matsuo R., Yamagata Y., Yoshimura A., Mochizuki T. (2012). Identification of QTL for aleurone traits contributing to lipid content of rice (Oryza sativa L.). Jpn. J. Crop Sci..

[bib60] Khin O.M., Sato M., Li-Tao T., Matsue Y., Yoshimura A., Mochizuki T. (2013). Close association between aleurone traits and lipid contents of rice grains observed in widely different genetic resources of Oryza sativa. Plant Prod. Sci..

[bib61] Kim Y.J., Yeu S.Y., Park B.S., Koh H.-J., Song J.T., Seo H.S. (2012). Protein Disulfide Isomerase-Like Protein 1-1 Controls Endosperm Development through Regulation of the Amount and Composition of Seed Proteins in Rice. PLoS One.

[bib62] Kim M.-S., Ko S.-R., Le V.T., Jee M.-G., Jung Y.J., Kang K.-K., Cho Y.-G. (2022). Development of SNP markers from GWAS for selecting seed coat and aleurone layers in brown rice (Oryza sativa L.).. Genes.

[bib63] Kumar S.B., Venkateswaran K., Kundu S. (2010). Alternative Conformational Model of a Seed Protein DeK1 for Better Understanding of Structure-Function Relationship. J. Protein Proteonomics.

[bib64] Lebert L., Buche F., Sorin A., Aussenac T. (2022). The Wheat Aleurone Layer: Optimisation of Its Benefits and Application to Bakery Products. Foods.

[bib65] Lee K.P., Piskurewicz U., Turecková V., Strnad M., Lopez-Molina L. (2010). A seed coat bedding assay shows that RGL2-dependent release of abscisic acid by the endosperm controls embryo growth in Arabidopsis dormant seeds. Proc. Natl. Acad. Sci. USA.

[bib66] Lefebvre V., North H., Frey A., Sotta B., Seo M., Okamoto M., Nambara E., Marion-Poll A. (2006). Functional analysis of Arabidopsis NCED6 and NCED9 genes indicates that ABA synthesized in the endosperm is involved in the induction of seed dormancy. Plant J..

[bib67] Legland D., Alvarado C., Badel E., Guillon F., King A., Le T.D.Q., Rivard C., Paré L., Chateigner-Boutin A.L., Girousse C. (2022). Synchrotron Based X-ray Microtomography Reveals Cellular Morphological Features of Developing Wheat Grain. Appl. Sci..

[bib68] Lewis D., Bacic A., Chandler P.M., Newbigin E.J. (2009). Aberrant cell expansion in the elongation mutants of barley. Plant Cell Physiol..

[bib69] Li X., Hu H., Xu F., Liu Z., Zhang L., Zhang H. (2019). Effects of aleurone-rich fraction on the hydration and rheological properties attributes of wheat dough. Int. J. Food Sci. Technol..

[bib70] Li D.Q., Wu X.B., Wang H.F., Feng X., Yan S.J., Wu S.Y., Liu J.X., Yao X.F., Bai A.N., Zhao H. (2021). Defective mitochondrial function by mutation in THICK ALEURONE 1 encoding a mitochondrion-targeted single-stranded DNA-binding protein leads to increased aleurone cell layers and improved nutrition in rice. Mol. Plant.

[bib71] Li B., Sun C., Li J., Gao C. (2024). Targeted genome-modification tools and their advanced applications in crop breeding. Nat. Rev. Genet..

[bib72] Lid S.E., Gruis D., Jung R., Lorentzen J.A., Ananiev E., Chamberlin M., Niu X., Meeley R., Nichols S., Olsen O.A. (2002). The defective kernel 1 (dek1) gene required for aleurone cell development in the endosperm of maize grains encodes a membrane protein of the calpain gene superfamily. Proc. Natl. Acad. Sci. USA.

[bib73] Lid S.E., Al R.H., Krekling T., Meeley R.B., Ranch J., Opsahl-Ferstad H.-G., Olsen O.-A. (2004). The maize disorganized aleurone layer 1 and 2 ( dil1, dil2) mutants lack control of the mitotic division plane in the aleurone layer of developing endosperm. Planta.

[bib74] Liu J., Wu X., Yao X., Yu R., Larkin P.J., Liu C.-M. (2018). Mutations in the DNA demethylase OsROS1 result in a thickened aleurone and improved nutritional value in rice grains. Proc. Natl. Acad. Sci. USA.

[bib75] Luh B.S., Barber S., Benedito de Barber C. (1991). Rice.

[bib76] McClintock B. (1950). The origin and behavior of mutable loci in maize. Proc. Natl. Acad. Sci. USA.

[bib77] Meziani S., Nadaud I., Tasleem-Tahir A., Nurit E., Benguella R., Branlard G. (2021). Wheat aleurone layer: A site enriched with nutrients and bioactive molecules with potential nutritional opportunities for breeding. J. Cereal. Sci..

[bib78] Miller K.B. (2020). Review of whole grain and dietary fiber recommendations and intake levels in different countries. Nutr. Rev..

[bib79] Minase M., Senjo O., Nagata Y. (1962). Studies on the Soft-Textured Rice Kernel : V. Thickness of bran layer. Jpn. J. Crop Sci..

[bib80] Miyoshi K., Kagaya Y., Ogawa Y., Nagato Y., Hattori T. (2002). Temporal and spatial expression pattern of the OSVP1 and OSEM genes during seed development in rice. Plant Cell Physiol..

[bib81] Morrison I.N., Kuo J., O’Brien T.P. (1975). Histochemistry and fine structure of developing wheat aleurone cells. Planta.

[bib82] Nagato K., Ebata M. (1960). Effects of Temperature in the Ripening Periods upon the Development and Qualities of Lowland Rice Kernels. Jpn. J. Crop Sci..

[bib83] Nagato K., Ebata M. (1965). Effects of High Temperature during Ripening Period on the Development and the Quality of Rice Kernels. Jpn. J. Crop Sci..

[bib84] Nagato K., Ebata M., Kishi Y. (1966). Effects of High Temperature during Ripening Period on the Qualities of Indica Rice. Jpn. J. Crop Sci..

[bib85] Nelson O.E., Chang M.T. (1974). Effect of Multiple Aleurone Layers on the Protein and Amino Acid Content of Maize Endosperm 1. Crop Sci..

[bib86] Nguyen T.M.P., Abiko T., Nakamura T., Mochizuki T. (2022). Development and Application of a Plate Method for Visualizing Aleurone Layers in Mature Rice Grains. Plant Prod. Sci..

[bib87] Olsen O.-A. (2001). ENDOSPERM DEVELOPMENT: Cellularization and Cell Fate Specification. Annu. Rev. Plant Physiol. Plant Mol. Biol..

[bib88] Olsen O.-A. (2004). Nuclear endosperm development in cereals and Arabidopsis thaliana. Plant Cell.

[bib89] Olsen O.A. (2020). The Modular Control of Cereal Endosperm Development. Trends Plant Sci..

[bib90] Olsen L.T., Divon H.H., Al R., Fosnes K., Lid S.E., Opsahl-Sorteberg H.G. (2008). The defective seed5 (des5) mutant: Effects on barley seed development and HvDek1, HvCr4, and HvSal1 gene regulation. J. Exp. Bot..

[bib91] Olsen O.A., Perroud P.F., Johansen W., Demko V. (2015). DEK1; missing piece in puzzle of plant development. Trends Plant Sci..

[bib92] Ono A., Yamaguchi K., Fukada-Tanaka S., Terada R., Mitsui T., Iida S. (2012). A null mutation of ROS1a for DNA demethylation in rice is not transmittable to progeny. Plant J..

[bib93] Opanowicz M., Hands P., Betts D., Parker M.L., Toole G.A., Mills E.N.C., Doonan J.H., Drea S. (2011). Endosperm development in Brachypodium distachyon. J. Exp. Bot..

[bib94] Pascoe D.A., Fulcher R.G. (2007). Whole Grains and Health.

[bib95] Paulsmeyer M.N., Juvik J.A. (2023). Increasing aleurone layer number and pericarp yield for elevated nutrient content in maize. G3 (Bethesda)..

[bib96] Penfield S., Rylott E.L., Gilday A.D., Graham S., Larson T.R., Graham I.A. (2004). Reserve mobilization in the Arabidopsis endosperm fuels hypocotyl elongation in the dark, is independent of abscisic acid, and requires phosphoenolpyruvate carboxykinase1. Plant Cell.

[bib97] Pfeifer M., Kugler K.G., Sandve S.R., Zhan B., Rudi H., Hvidsten T.R., Mayer K.F.X., Olsen O.-A., International Wheat Genome Sequencing Consortium (2014). Genome interplay in the grain transcriptome of hexaploid bread wheat. Science.

[bib98] Pu C.-X., Ma Y., Wang J., Zhang Y.-C., Jiao X.-W., Hu Y.-H., Wang L.-L., Zhu Z.-G., Sun D., Sun Y. (2012). Crinkly4 receptor-like kinase is required to maintain the interlocking of the palea and lemma, and fertility in rice, by promoting epidermal cell differentiation. Plant J..

[bib99] Qi X., Li S., Zhu Y., Zhao Q., Zhu D., Yu J. (2017). ZmDof3, a maize endosperm-specific Dof protein gene, regulates starch accumulation and aleurone development in maize endosperm. Plant Mol. Biol..

[bib100] Rahman S., Copeland L., Atwell B.J., Roberts T.H. (2022). Impact of elevated atmospheric CO2 on aleurone cells and starch granule morphology in domesticated and wild rices. J. Cereal. Sci..

[bib101] Ren Z.-W., Yang M., McKenna B.A., Lian X.-M., Zhao F.-J., Kopittke P.M., Lombi E., Wang P. (2023). Fast X-ray fluorescence microscopy provides high-throughput phenotyping of element distribution in seeds. Plant Physiol..

[bib102] Rosa-Sibakov N., Poutanen K., Micard V. (2015). How does wheat grain, bran and aleurone structure impact their nutritional and technological properties?. Trends Food Sci. Technol..

[bib144] Royo J., Gómez E., Sellam O., Gerentes D., Paul W., Hueros G. (2014). Two maize END-1 orthologs, BETL9 and BETL9like, are transcribed in a non-overlapping spatial pattern on the outer surface of the developing endosperm. Front. Plant Sci..

[bib103] Seo S.W., Chamura S. (1980). Occurrence of Varietal Differences in Prorein, Phosphorus, and Potassium Content in Brown Rice, and Influence of Temperature and Shading during the Ripening Period on It. Jpn. J. Crop Sci..

[bib104] She Y., Gao X., Lu W.C., Yang Z., Niu B., Zhou Y., Huang X.Y., Chen C. (2025). Ionomic and metabolomic analyses reveal association between nutritional value and aleurone layer thickness in rice. Food Chem..

[bib105] Shen B., Li C., Min Z., Meeley R.B., Tarczynski M.C., Olsen O.A. (2003). Sal1 determines the number of aleurone cell layers in maize endosperm and encodes a class E vacuolar sorting protein. Proc. Natl. Acad. Sci. USA.

[bib106] Shi K., Yin T., Zhu Y., Liu B., Tang L., Cao W., Liu L. (2022). Estimating the effect of low-temperature stress on the spatial distribution patterns of protein in wheat grains. J. Cereal. Sci..

[bib107] Suzuki M., Latshaw S., Sato Y., Settles A.M., Koch K.E., Hannah L.C., Kojima M., Sakakibara H., McCarty D.R. (2008). The Maize Viviparous8 Locus, Encoding a Putative ALTERED MERISTEM PROGRAM1-Like Peptidase, Regulates Abscisic Acid Accumulation and Coordinates Embryo and Endosperm Development. Plant Physiol..

[bib108] Taira H., Taira H., Fujii K. (1979). Influence of Cropping Season on Lipid Content and Fatty Acid Composition of Lowland Non-glutious Brown Rice. Jpn. J. Crop Sci..

[bib109] Takafuji Y., Shimizu-Sato S., Ta K.N., Suzuki T., Nosaka-Takahashi M., Oiwa T., Kimura W., Katoh H., Fukai M., Takeda S. (2021). High-resolution spatiotemporal transcriptome analyses during cellularization of rice endosperm unveil the earliest gene regulation critical for aleurone and starchy endosperm cell fate specification. J. Plant Res..

[bib110] Teas H.J. (1952). A Morphological Basis for Higher Niacin in Sugary Maize. Proc. Natl. Acad. Sci. USA.

[bib111] Tian Q., Olsen L., Sun B., Lid S.E., Brown R.C., Lemmon B.E., Fosnes K., Gruis D.F., Opsahl-Sorteberg H.G., Otegui M.S., Olsen O.A. (2007). Subcellular localization and functional domain studies of DEFECTIVE KERNEL1 in maize and Arabidopsis suggest a model for aleurone cell fate specification involving CRINKLY4 and SUPERNUMERARY ALEURONE LAYER1. Plant Cell.

[bib112] Tran D., Galletti R., Neumann E.D., Dubois A., Sharif-Naeini R., Geitmann A., Frachisse J.M., Hamant O., Ingram G.C. (2017). A mechanosensitive Ca2+ channel activity is dependent on the developmental regulator DEK1. Nat. Commun..

[bib113] Wang C., Barry J.K., Min Z., Tordsen G., Rao A.G., Olsen O.A. (2003). The Calpain Domain of the Maize DEK1 Protein Contains the Conserved Catalytic Triad and Functions as a Cysteine Proteinase. J. Biol. Chem..

[bib114] Wang Z., Gu Y.J., Hirasawa T., Ookawa T., Yanahara S. (2004). Comparison of Caryopsis development between two rice varieties with remarkable difference in grain weights. Acta Bot. Sin..

[bib115] Wang X., Zhou W., Lu Z., Ouyang Y., Yao J. (2015). A lipid transfer protein, OsLTPL36, is essential for seed development and seed quality in rice. Plant Sci..

[bib116] Wang F.X., Shang G.D., Wu L.Y., Xu Z.G., Zhao X.Y., Wang J.W. (2020). Chromatin Accessibility Dynamics and a Hierarchical Transcriptional Regulatory Network Structure for Plant Somatic Embryogenesis. Dev. Cell.

[bib117] Wei S., Guo B., Feng L., Jiang T., Li M., Wei Y. (2017). Cadmium distribution and characteristics of cadmium-binding proteins in rice (Oryza sativa L.) kernel. Food Sci. Technol. Res..

[bib118] Wolf M.J., Cutler H.C., Zuber M.S., Khoo U. (1972). Maize with Multilayer Aleurone of High Protein Content 1. Crop Sci..

[bib119] Wu H., Becraft P.W. (2021). Comparative transcriptomics and network analysis define gene coexpression modules that control maize aleurone development and auxin signaling. Plant Genome.

[bib120] Wu X., Liu J., Li D., Liu C.-M. (2016). Rice caryopsis development I: Dynamic changes in different cell layers. J. Integr. Plant Biol..

[bib121] Wu X., Liu J., Li D., Liu C.-M. (2016). Rice caryopsis development II: Dynamic changes in the endosperm. J. Integr. Plant Biol..

[bib122] Wu H., Gontarek B.C., Yi G., Beall B.D., Neelakandan A.K., Adhikari B., Chen R., McCarty D.R., Severin A.J., Becraft P.W. (2020). The thick aleurone1 gene encodes a NOT1 subunit of the CCR4-NOT complex and regulates cell patterning in endosperm. Plant Physiol..

[bib123] Wu H., Becraft P.W., Dannenhoffer J.M. (2022). Maize Endosperm Development: Tissues, Cells, Molecular Regulation and Grain Quality Improvement. Front. Plant Sci..

[bib124] Xiong F., Yu X.R., Zhou L., Wang Z., Wang F., Xiong A.S. (2013). Structural development of aleurone and its function in common wheat. Mol. Biol. Rep..

[bib125] Xu G., Zhang X. (2023). Mechanisms controlling seed size by early endosperm development. Suo (Helsinki).

[bib126] Xu X.E.Z., Zhang D., Yun Q., Zhou Y., Niu B., Chen C. (2021). OsYUC11 -mediated auxin biosynthesis is essential for endosperm development of rice. Plant Physiol..

[bib127] Xu D., Dondup D., Dou T., Wang C., Zhang R., Fan C., Guo A., Lhundrup N., Ga Z., Liu M. (2023). HvGST plays a key role in anthocyanin accumulation in colored barley. Plant J..

[bib128] Xu Y., Chen S., Xue M., Chen X., Liu Z., Wei X., Gao J.-P., Chen C. (2023). Mapping and validation of quantitative trait loci associated with dorsal aleurone thickness in rice (Oryza sativa). Theor. Appl. Genet..

[bib129] Yan L.L., Mi J., Shen C.C., Qian R., Wang J., Pu C.X., Sun Y. (2023). OsCIP1, a secreted protein, binds to and stabilizes OsCR4 to promote aleurone layer development, seed germination and early seedling growth in rice. Plant Sci..

[bib130] Yi G., Lauter A.M., Scott M.P., Becraft P.W. (2011). The thick aleurone1 mutant defines a negative regulation of maize aleurone cell fate that functions downstream of defective kernel. Plant Physiol..

[bib131] Yi G., Neelakandan A.K., Gontarek B.C., Vollbrecht E., Becraft P.W. (2015). The naked endosperm genes encode duplicate INDETERMINATE domain transcription factors required for maize endosperm cell patterning and differentiation. Plant Physiol..

[bib145] Yu R., Wu X., Liu J. (2021). Rice with multilayer aleurone: a larger sink for multiple micronutrients. Rice (N Y).

[bib132] Yuan Y., Huo Q., Zhang Z., Wang Q., Wang J., Chang S., Cai P., Song K.M., Galbraith D.W., Zhang W. (2024). Decoding the gene regulatory network of endosperm differentiation in maize. Nat. Commun..

[bib133] Zhan J., Thakare D., Ma C., Lloyd A., Nixon N.M., Arakaki A.M., Burnett W.J., Logan K.O., Wang D., Wang X. (2015). RNA sequencing of laser-capture microdissected compartments of the maize kernel identifies regulatory modules associated with endosperm cell differentiation. Plant Cell.

[bib134] Zhang H., Lang Z., Zhu J.K. (2018). Dynamics and function of DNA methylation in plants. Nat. Rev. Mol. Cell Biol..

[bib135] Zhang H., Xiao Y., Deng X., Feng H., Li Z., Zhang L., Chen H. (2020). OsVPE3 Mediates GA-induced Programmed Cell Death in Rice Aleurone Layers via Interacting with Actin Microfilaments. Rice.

[bib136] Zhang J., Niu B., E Z., Chen C. (2021). Towards Understanding the Genetic Regulations of Endosperm Development in Rice. Chin. J. Rice Sci..

[bib137] Zhao D., Chen S., Han Y., Liu G., Liu J., Yang Q., Zhang T., Shen J., Fan X., Zhang C. (2024). A CRISPR/Cas9-mediated mutant library of seed-preferred genes in rice. Plant Biotechnol. J..

[bib138] Zheng X., Li Q., Li C., An D., Xiao Q., Wang W., Wu Y. (2019). Intra-Kernel Reallocation of Proteins in Maize Depends on VP1-Mediated Scutellum Development and Nutrient Assimilation. Plant Cell.

[bib139] Zhou S., Li X., Liu Q., Zhao Y., Jiang W., Wu A., Zhou D.X. (2021). DNA demethylases remodel DNA methylation in rice gametes and zygote and are required for reproduction. Mol. Plant.

[bib140] Zhou T., Ying R., Huang M., Tang Z. (2022). Effect of aleurone-rich fraction on texture and nutritional properties of breads. Int. J. Food Sci. Technol..

[bib141] Zhou H., Deng X.W., He H. (2023). Gene expression variations and allele-specific expression of two rice and their hybrid in caryopses at single-nucleus resolution. Front. Plant Sci..

[bib142] Tianshun Z., Dong Y., Liubing W., Yusheng X., Meijuan D., Dingyang Y. (2024). Seed Storability in Rice: Physiological Foundations, Molecular Mechanisms, and Applications in Breeding. Rice Sci..

[bib143] Zhu C., Kobayashi K., Loladze I., Zhu J., Jiang Q., Xu X., Liu G., Seneweera S., Ebi K.L., Drewnowski A. (2018). Carbon dioxide (CO2) levels this century will alter the protein, micronutrients, and vitamin content of rice grains with potential health consequences for the poorest rice-dependent countries. Sci. Adv..

